# How do plant‐based milks compare to cow's milk nutritionally? An audit of the plant‐based milk products available in Australia

**DOI:** 10.1111/1747-0080.12906

**Published:** 2024-09-29

**Authors:** Isobel Harmer, Joel C. Craddock, Karen E. Charlton

**Affiliations:** ^1^ School of Medical, Indigenous and Health Sciences, Faculty of Science Medicine and Health University of Wollongong Wollongong New South Wales Australia

**Keywords:** audit, calcium, fortification, iodine, plant‐based milk, supermarket

## Abstract

**Aim:**

This cross‐sectional study aims to explore the nutritional composition, cost, country of origin and fortification status of plant‐based milk products available for purchase in Illawarra supermarkets and make various comparisons between types of plant‐based milks and cow's milk.

**Methods:**

Plant‐based milk information was collected from nutrition information panels on packaging and manufacturer websites. Product ingredient lists, including fortifiers, were analysed to estimate the nutrient composition of the identified plant‐based milks, including nutrients beyond those listed by manufacturers. Descriptive statistics were used to summarise the characteristics of the plant‐based milks identified in the audit. For non‐normally distributed data, a Kruskal–Wallis H test with pairwise multiple comparisons and a Bonferroni adjustment were undertaken to explore the differences between various types of plant‐based milk and cow's milk.

**Results:**

One hundred twenty‐nine plant‐based milk products were identified in the audit, primarily almond, oat and soy‐based beverages. Of these, 80.6% were fortified with calcium; however, fortification with other micronutrients was less common, ranging from 27.1% being fortified with vitamin B12 and 3.1% being fortified with iodine. The median plant‐based milk cost was AU$3.5/L (Q1–Q3: AU$2.8–4.5/L) and 87.6% of products were Australian made. Overall, particularly due to low fortification rates, plant‐based milks identified in the audit had significantly lower levels of protein, sugar, iodine, phosphorus, zinc and vitamins A, B2 and B12 compared to cow's milk. However, there was no significant difference in protein content between soy milk and cow's milk.

**Conclusions:**

The nutritional content of plant‐based milks identified in this audit varied, and in most instances, Australian plant‐based milks were found to be nutritionally different to cow's milk.

## 1 INTRODUCTION

Plant‐based milk alternatives are comprised of plant matter that has been processed, aiming to create a final product that imitates the flavour, texture and appearance of cow's milk.^1^ Plant‐based milks have existed in Australian supermarkets since the 1980s and have become increasingly popular in recent years.^2^, ^3^, ^4^ The Australian Bureau of Statistics estimates a 4.2 g per day per person, or 38%, increase in plant‐based milk consumption between the 2018–2019 and 2022–2023 financial years.^5^ Additionally there was an estimated simultaneous decrease in cow's milk consumption,^5^ suggesting a potential replacement of cow's milk with plant‐based milk. The Australian plant‐based milk market generated AU$464.6 million in revenue from 2022 to 2023 with the most popular plant‐based milk beverages being soy and almond‐based.^4^ Alternatives, such as oat milk, are becoming more prevalent.^4^

Dairy products including cow's milk are a core food group of Australian diets, providing a wide range of essential nutrients. An analysis of the Australian National Nutrition and Physical Activity Survey 2011–2012 data reported that cow's milk provided more than 5% of total daily intake for many nutrients, namely calcium (21.1%), vitamin B12 (20.7%), iodine (19.3%), riboflavin (15.8%), phosphorus (10%), saturated fat (8.4%), potassium (8.1%), vitamin A (6.4%), protein (6.1%), vitamin B6 (5.7%), zinc (5.2%), magnesium (5.1%) and total fat (5%).^6^ This data shows that cow's milk is a primary provider of many essential nutrients in the Australian diet.

Considering the contribution of nutrients provided by cow's milk in the Australian diet, together with the shift away from traditional cow's milk towards plant‐based milk by some consumers, characterising the nutritional composition of plant‐based milks available for sale is crucial. Compared to cow's milk, unfortified plant‐based milks have lower saturated fat, energy and micronutrients including calcium, zinc, iodine, phosphorus and vitamins A, D, B2 and B12.^1^, ^7^, ^8^, ^9^, ^10^, ^11^, ^12^, ^13^, ^14^ Further, protein content is lower in all unfortified plant‐based milk types except for legume‐based soy and pea beverages.^1^, ^7^, ^8^, ^9^, ^10^, ^11^, ^12^, ^13^, ^14^ However, Food Standards Australia and New Zealand (FSANZ) does not currently have a food standard on what constitutes a plant‐based milk in terms of composition and it is not mandatory for plant‐based milks to be fortified with any nutrients.^15^ Therefore, the micronutrient content of plant‐based milks in Australia varies depending on fortification by manufacturers.

There is limited information on the fortification status of plant‐based milks available for sale in Australia. In an audit of products available in metropolitan Australia conducted between November 2019 and January 2020, only 57% of plant‐based milks were fortified with any nutrient, while none were fortified with iodine or zinc.^8^ Additionally, Craig et al. in 2021 reported that 79% of plant‐based milk beverages were fortified with calcium, 21% with vitamin D and 35% with vitamin B12.^13^ The disparity between these studies may suggest a change in fortification over time; however, the range of plant‐based milks assessed by Craig et al. was likely limited as the research was conducted online and included products for sale in only one supermarket (Woolworths).

There is also limited information on the nutritional composition of plant‐based milks in the Australian context, particularly in comparison to cow's milk. Australian food composition databases do reference various plant‐based milk types and fortification statuses, with the 2022 Australian Food Composition Database having additional plant‐based products compared to the 2011–2013 AUSNUT database.^16^, ^17^ However, a 2024 study found that more than two thirds of dairy alternatives identified in Australian supermarkets were not accurately reflected (within a 10% error range) in the Australian Food Composition Database (2022).^18^ That is, the current available Australian food composition databases do not accurately represent the nutrient content of the rapidly expanding Australian plant‐based milk market. Alternatively, an audit of plant‐based milks conducted in metropolitan Australia in early 2020 investigated the nutritional composition of plant‐based milks, and made comparison to cow's milk.^8^ The plant‐based milks identified were found to contain significantly lower amounts of protein, zinc, phosphorus and vitamins A, B2 and B12 compared to cow's milk. Furthermore, iodine was significantly lower than cow's milk in all plant‐based milk categories except grain‐based beverages, where median iodine levels were only a fifth of that found in cow's milk.^8^ To the authors' knowledge, no other previous Australian research has thoroughly investigated the nutritional composition of plant‐based milk compared to cow's milk.

Given the rising popularity of plant‐based milks and rapidly expanding Australian plant‐based milk market,^4^ it is timeous to provide an update of the nutritional composition of plant‐based milks available for purchase by Australian consumers to identify any potential nutrient deficits associated with direct replacement of cow's milk with plant‐based alternatives. This study aims to explore the nutritional composition, fortification, cost, country of origin and fortification status of plant‐based milk products available for purchase in Illawarra supermarkets (a regional area of Southeast Australia). This study additionally aims to compare plant‐based milk nutrient composition to that of cow's milk.

## 2 METHODS

The audit, conducted in April 2023, aimed to capture information from both major supermarket chains and local food retailers located within the Illawarra region of NSW. This region was chosen for data collection convenience. The Illawarra region was defined as the local government areas of Wollongong, Shellharbour and Kiama (Appendix 1). The websites of the four major supermarket retailers in Australia (Woolworths, Coles, Aldi and IGA) were searched to identify stores located within the defined area.^19^ The stores were then sorted by Index of Relative Socioeconomic Advantage and Disadvantage (IRSAD) quintiles at the statistical area level 2, a metric used to assess the socioeconomic status of a given area that has been developed by the Australian Bureau of Statistics.^20^ After sorting according to IRSAD quintiles, three stores from each quintile were randomly selected providing a total of 15 major supermarkets to audit across the five socioeconomic ranked locations.

To uncover the smaller food retailers located within the Illawarra, the results from a Google maps search for ‘health food store’, ‘supermarket’ and ‘grocery store’ were collated.^21^ Stores described as selling nutritional supplements and those advertised as being specifically ethnicity‐focussed were excluded. These smaller food retailers were also sorted into IRSAD quintiles at statistical area level 2. Since these smaller food retailers make up less than 20% of the food retail market in Australia,^19^ one food retailer from each quintile was randomly selected for the audit.

A member of the research team attended each selected store and identified all plant‐based milks on shelves. Manufacturer websites were also utilised to extract further information, where available. Plant‐based milks were identified as any products sold with the intention of being a cow's milk alternative within the refrigerated and long‐life milk sections of the supermarket. Flavoured beverages were excluded and items such as tinned coconut milk, although labelled as ‘milk’, are not intended for consumption as fluid milk and were not sampled. A photograph of each side of the package for every plant‐based milk was captured for further analysis.

The country‐of‐origin information for each plant‐based milk product was obtained through the photographed packaging and recorded in a Microsoft Excel spreadsheet. To capture data on the cost of plant‐based milks, the full price (non‐discounted price) of each milk at every audited supermarket was collated. Data on the nutritional composition of the plant‐based milks was recorded in the spreadsheet for the nutrients required by law to be displayed on the nutrition information panel (energy, protein, carbohydrate, sugars, total fats, saturated fats and sodium). Additional nutrients listed by manufacturers were also recorded, including vitamins or minerals listed in the ingredients list, for products that had been fortified. In order to quantify nutrients of interest to the current study, such as calcium, iodine and zinc, secondary ingredient analysis was undertaken following methodology previously used in plant‐based milk investigations in Australia and the UK.^8^, ^22^

For the secondary analysis, the ingredients list provided on the plant‐based milk packaging was entered as a new recipe into the Foodworks dietary assessment software package,^23^ and analysed using the AUSNUT 2011–2013 food composition database.^17^ Characterising ingredients were only analysed as FSANZ does not require manufacturers to list the amount, or percentage contribution, of ingredients other than characterising ingredients. Characterising ingredients are defined as ‘an ingredient or a category of ingredients of the food that: (a) is mentioned in the name of the food; or (b) is usually associated with the name of the food by a consumer; or (c) is emphasised on the label of the food in words, pictures or graphics’.^24^ In the case of plant‐based milks, this would indicate almonds as a characterising ingredient for ‘almond milk’ or soy as a characterising ingredient for ‘soy beverage’. This analysis provided a *minimum estimation* of micronutrients per 100 mL of plant‐based milk that have previously been identified to be lower in plant‐based milk than cow's milk but are not consistently displayed in the nutrition information panel, namely calcium, iodine, zinc, phosphorus, vitamin A (as retinol equivalents), vitamin B2 and vitamin B12.^1^, ^7^, ^8^, ^9^, ^10^, ^11^, ^12^, ^13^, ^14^ Nutrient information from secondary analysis was gathered only to supplement the data collected from the nutrition information panel. That is, if the nutrient value was already listed in the nutrition information panel, the value estimated through the secondary analysis was not utilised.

At the time of this analysis, vitamin D values were not available in the AUSNUT 2011–2013 database, therefore only plant‐based milks that had vitamin D data on their nutrition information panel had a value recorded against their product. As hemp seeds were not available within the AUSNUT 2011–2013 database, their nutritional composition was obtained through the United States Department of Agriculture Standard Reference Legacy database (2018).^25^ The two pea‐protein based beverages that were found were not analysed as there was no data for pea‐protein in the AUSNUT 2011–2013 database nor identified in other equivalent databases. To compare the nutrient composition of plant‐based milks with that of cow's milk, a selection of seven cow's milk items, including those of various fat contents and lactose status, were selected from the AUSNUT 2011–2013 database and averaged (Appendix 2).^17^

Prior to analysis, another member of the research team who is an Accredited Practising Dietitian reviewed a 10% sample of collected data and compared it to the source material (manufacturer websites and product photographs) as a quality check. An error was defined as an individual number entered in the spreadsheet that did not match the source material. The error rate was calculated as the total number of errors divided by the total number of data points multiplied by 100. The error rate was below 5% and thus deemed appropriate to continue onto statistical analysis, guided by the Guideline for Good Clinical Practice by the International Council for Harmonisation of Technical Requirements for Pharmaceuticals for Human Use.^26^

Data was analysed using IBM SPSS Statistics for Windows software (Version 29.0.1.0 (171) Armonk, NY: IBM Corp). Descriptive statistics were used to summarise the characteristics of the plant‐based milks identified in the audit. Normality was tested using the Shapiro–Wilk test. For non‐normally distributed data, a Kruskal–Wallis H test with pairwise multiple comparisons and a Bonferroni adjustment were undertaken to explore the differences between various types of plant‐based milk and cow's milk. Non‐parametric data was presented as median and interquartile range. Significance was set at *p* < 0.05. The reporting of results is compliant with strengthening the reporting of observational studies in epidemiology (STROBE) statement guidelines.^27^

## 3 RESULTS

The audit identified 129 unique plant‐based milk products that included almond, oat, soy, coconut, rice cashew, hazelnut, macadamia, pea and hemp‐based beverages (Table 1). There were also five products that included a combination of plant materials. The three most common types of plant‐based milk were almond, oat and soy beverages which comprised 82.1% of all products identified. The other 17.9% included all other varieties, but primarily coconut and rice‐based beverages, each comprising 3.9% of all products. Various combinations, such as pea and hemp‐based plant‐based milks, were minimal and thus were respectively grouped into ‘blends’ and ‘other’. Overall, 87.6% of plant‐based milks were made in Australia. Of the Australian‐made plant‐based milks, on average 92.9% of ingredients were of Australian origin.

**TABLE 1:** Table displaying the number of products available for purchase of each milk type.

Plant‐based milk type	Number of products, *n* (%)
Almond	40 (31.0%)
Oat	39 (30.2%)
Soy	27 (20.9%)
Coconut	5 (3.9%)
Rice	5 (3.9%)
Other nuts (cashew, hazelnut, macadamia)	5 (3.9%)
Blends^a^	5 (3.9%)
Other^b^	3 (2.3%)
Total	129 (100%)

^a^ Plant‐based milks comprised of two or more plant materials.

^b^ Pea and hemp‐based plant‐based milks.

As shown in Table 2, plant‐based milks were most consistently fortified with calcium (80.6% of products). All beverages that were fortified with only a single nutrient were fortified with calcium. The next most commonly fortified nutrient was vitamin B12 (27.1% of products), followed by vitamin B2 (24.8%), vitamin D (17.8%) and phosphorus (10.1%). Only 3.1% of plant‐based milks were fortified with iodine or magnesium. Soy beverages tended to be the most consistently fortified whereas none of the ‘other’ plant‐based milks were fortified with any micronutrients.

**TABLE 2:** The frequency (percent) of products within each plant‐based milk type that are fortified with different micronutrients.

Type	Micronutrient								
	Calcium, *n* (%)	Vitamin B12, *n* (%)	Vitamin B2, *n* (%)	Vitamin D, *n* (%)	Vitamin A, *n* (%)	Phosphorus, *n* (%)	Magnesium, *n* (%)	Iodine, *n* (%)	Total products in category, *n*
Almond	32 (80.0%)	8 (20.0%)	7 (17.5%)	3 (7.5%)	0 (0.0%)	3 (7.5%)	0 (0.0%)	0 (0.0%)	40
Oat	32 (82.1%)	11 (28.2%)	9 (23.1%)	12 (30.8%)	2 (5.1%)	3 (7.7%)	0 (0.0%)	4 (10.3%)	39
Soy	25 (92.6%)	13 (48.2%)	13 (48.2%)	7 (25.9%)	13 (48.2%)	6 (22.2%)	4 (14.8%)	0 (0.0%)	27
Coconut	3 (60.0%)	0 (0.0%)	0 (0.0%)	0 (0.0%)	0 (0.0%)	1 (20.0%)	0 (0.0%)	0 (0.0%)	5
Rice	4 (80.0%)	0 (0.0%)	0 (0.0%)	0 (0.0%)	0 (0.0%)	0 (0.0%)	0 (0.0%)	0 (0.0%)	5
Other nuts	5 (100.0%)	1 (20.0%)	1 (20.0%)	0 (0.0%)	0 (0.0%)	0 (0.0%)	0 (0.0%)	0 (0.0%)	5
Blends^a^	3 (60.0%)	2 (40.0%)	2 (40.0%)	1 (20.0%)	0 (0.0%)	0 (0.0%)	0 (0.0%)	0 (0.0%)	5
Other^b^	0 (0.0%)	0 (0.0%)	0 (0.0%)	0 (0.0%)	0 (0.0%)	0 (0.0%)	0 (0.0%)	0 (0.0%)	3
Total	104 (80.6%)	35 (27.1%)	32 (24.8%)	23 (17.8%)	15 (11.6%)	13 (10.1%)	4 (3.1%)	4 (3.1%)	129

^a^ Plant‐based milks comprised of two or more plant materials.

^b^ Pea and hemp‐based plant‐based milks.

The median plant‐based milk cost was AU$3.5/L (Q1–Q3: AU$2.8–4.5/L) within Illawarra supermarkets (Table 3). Soy milk was the cheapest with a median cost of AU$2.5/L (Q1–Q3: AU$1.8–3.2/L) whereas oat and ‘other’ beverages were the most expensive with a median cost of AU$4.4 (Q1–Q3: AU$3.5–5.0/L) and AU$4.9 (range: AU$1.5–6.7/L). There was no statistically significant difference between fortification status and plant‐based milk cost, when comparing unfortified plant‐based milks (4.3 [2.5–5.3] $AU/L) with beverages fortified either with one (3.5 [2.6–4.5] $AU/L) or two or more micronutrients (3.4 [2.9–4.1] $AU/L).

**TABLE 3:** The price of each type of plant‐based milk.

Type	Median price (AU$/L) (Q1–Q3)
Soy (*n* = 27)	2.5 (1.8–3.2)
Rice (*n* = 5)	2.6 (2.0–3.3)^a^
Coconut (*n* = 5)	3.2 (2.3–3.7)^a^
Almond (*n* = 40)	3.4 (2.8–4.4)
Blends (*n* = 5)	4.0 (2.9–4.0)^a^
Other nuts (*n* = 5)	4.1 (3.4–4.3)^a^
Oat (*n* = 39)	4.4 (3.5–5.0)
Other (*n* = 3)	4.9 (1.5–6.7)^a^
Total (*n* = 129)	3.5 (2.8–4.5)

^a^ Due to small group sizes median (minimum‐maximum) is displayed.

Regarding macronutrient content for different plant‐based milks compared to cow's milk (Table 4), there were no significant differences found for energy or carbohydrate content. Soy had a higher total fat content (3.0 *v* 1.2 g/100 mL, *p* < 0.05) whereas almond and rice milks were lower in saturated fat (0.2 and 0.1 *v* 0.8 g/100 mL, respectively, *p* < 0.05). Almond, coconut, oat, rice and other nut‐based plant‐based milks contained significantly lower protein content than cow's milk, with median values of 0.7 g (almond), 0.3 g (coconut), 1.0 g (oat), 0.2 g (rice) and 0.5 g (other) per 100 mL versus 3.6 g per 100 mL for cow's milk (*p* < 0.01). Almond, soy, other nuts and other plant‐based milks contained significantly less total sugar when compared to cow's milk, with median values ranging from 1.2 to 1.7 g per 100 mL versus 5.0 g per 100 mL for cow's milk (*p* < 0.01).

**TABLE 4:** Median (Q1–Q3) macronutrient content of different plant‐based milk types (combined fortified and unfortified beverages) compared to cow's milks.

Milk type	Energy (kJ/100 mL)	Protein (g/100 mL)	Total fat (g/100 mL)	Saturated fat (g/100 mL)	Carbohydrates (g/100 mL)	Total sugars (g/100 mL)
Cow's milk	191.0 (160.0–267.0)	3.60 (3.4–4.1)	1.2 (0.1–3.4)	0.8 (0.1–2.2)	5.0 (4.8–5.4)	5.0 (4.8–5.4)
Almond	116.5 (86.0–161.8)	0.7 (0.6–0.8)**	1.8 (1.4–2.5)	0.2 (0.1–0.2)*	1.7 (0.4–3.1)	1.3 (0.1–1.6)**
Coconut	184.0 (88.5–217.9)	0.30 (0.13–0.48)**	1.8 (1.8–2.5)	1.7 (1.5–2.3)	6.6 (0.5–7.6)	2.3 (0.2–3.5)
Oat	210.0 (193.0–250.0)	1.0 (0.6–1.1)**	2.0 (1.5–2.8)	0.3 (0.2–0.3)	6.7 (5.6–7.7)	2.7 (2.0–3.6)
Rice	200.0 (185.0–240.0)	0.2 (0.2–0.6)**	1.2 (0.8–1.2)	0.1 (0.1–0.1)*	9.0 (8.0–11.7)	3.6 (3.2–5.8)
Soy	246.0 (220.0–263.0)	3.2 (3.0–3.4)	3.0 (2.1–3.3)*	0.4 (0.3–0.4)	4.8 (2.8–5.1)	1.8 (1.6–2.2)**
Other nuts	125.0 (83.5–158.0)	0.5 (0.4–0.5)**	2.0 (1.7–2.9)	0.3 (0.3–0.3)	2.5 (0.7–2.6)	1.7 (0.2–1.9)**
Blends^a^	176.0 (122.5–217.5)	0.8 (0.5–3.3)	1.5 (1.5–1.9)	0.3 (0.2–1.0)	6.1 (1.6–8.0)	2.0 (1.1–2.8)
Other^b^	192.0 (108.0–298.0)	1.0 (0.1–1.6)	2.7 (1.4–3.3)	0.8 (0.3–2.0)	1.8 (0.9–1.8)	1.2 (0.2–1.2)**

^a^ Plant‐based milks comprised of two or more plant materials.

^b^ Pea and hemp‐based plant‐based milks.

** Indicates statistically significant (*p* < 0.01) difference compared to cow's milk using a Kruskal–Wallis pairwise multiple comparisons test with Bonferroni adjustment.

* Indicates statistically significant (*p* < 0.05) difference compared to cow's milk using a Kruskal–Wallis pairwise multiple comparisons test with Bonferroni adjustment.

Table 5 shows the micronutrient content of the audited plant‐based milks, with both fortified and unfortified beverages combined for analysis. Almond, soy, other nut and blended products contained significantly less iodine than cow's milk with median values ranging from 0.21 to 0.24 μg per 100 mL versus 22.3 μg per 100 mL for cow's milk (*p* < 0.01). All plant‐based milk varieties contained sodium and calcium in levels that were not significantly different to cow's milk. All plant‐based milks contained significantly lower levels of vitamin B12 than cow's milk, with a median vitamin B12 concentration of 0.00 μg per 100 mL for all plant‐based milk categories versus 0.60 μg per 100 mL for cow's milk (*p* < 0.05). Coconut, oat, other nuts and rice plant‐based milks were lower in vitamin B2 compared to cow's milk (median values ranging 0.00 to 0.01 *v* 0.20 mg/100 mL for cow's milk, *p* < 0.05), and almond, coconut and other nuts were lower in phosphorus (18.72, 2.93 and 10.85 v 96.00 mg/100 mL, respectively, *p* < 0.01). Almond, coconut and other nut products had lower amounts of zinc (0.12, 0.03, 0.08 v 0.34 mg/100 mL, respectively, *p* < 0.05) and only oat‐based beverages had significantly lower amounts of vitamin A compared to cow's milk (0.00 v 14.00 μg/100 mL, *p* < 0.01).

**TABLE 5:** Median (Q1–Q3) micronutrient content of different plant‐based milk types (combined fortified and unfortified beverages) compared to cow's milks.

Milk type	Sodium (mg/100 mL)	Calcium (mg/100 mL)	Iodine (μg/100 mL)	Zinc (mg/100 mL)	Phosphorus (mg/100 mL)	Vitamin A (μg/100 mL)	Vitamin B2 (mg/100 mL)	Vitamin B12 (μg/100 mL)
Cow's milk	46.00 (36.00–49.00)	118.00 (110.00–122.00)	22.30 (14.40–25.70)	0.34 (0.32–0.40)	96.00 (88.00–98.00)	14.00 (00.0–51.00)	0.20 (0.18–0.20)	0.60 (0.60–0.60)
Almond	39.50 (36.0–45.75)	100.00 (38.75–120.00)	0.21 (0.21–0.22)**	0.12 (0.09–0.16)*	18.72 (12.00–42.96)**	0.07 (0.05–0.08)	0.06 (0.04–0.13)	0.00 (0.00–0.00)**
Coconut	45.00 (33.00–53.50)	96.00 (1.41–120.00)	0.24 (0.22–0.25)	0.03 (0.03–0.04)**	2.93 (2.28–3.46)**	0.00 (0.00–0.00)	0.00 (0.00–0.00)**	0.00 (0.00)**
Oat	43.00 (40.00‐50.00)	120.00 (6.55–120.00)	0.61 (0.61–82)	0.23 (0.21–0.25)	34.00 (32.30–42.52	0.00 (0.00–0.00)**	0.00 (0.00–0.00)**	0.00 (0.00–0.21)**
Rice	65.00 (65.00–69.50)	120.00 (40.95–120)	0.24 (0.24–0.25)	0.25 (0.21–0.29)	36.88 (34.26–43.68)	0.00 (0.00–0.00)	0.01 (0.01–0.01)**	0.00 (0.00–0.00)**
Soy	60.00 (40.00–98.00)	120.00 (110.00–120.00)	0.24 (0.23–0.25)**	0.51 (0.05–0.60)	72.8 (17.16–84.0)	0.17 (0.13–44.00)	0.03 (0.02–0.20)	0.00 (0.00–0.32)**
Other nuts	42.00 (40.00–57.00)	120.00 (41.2–120.00)	0.21 (0.21–0.21)**	0.08 (0.04–0.13)*	10.85 (5.50–33.43)**	0.03 (0.00–0.11)	0.01 (0.00–0.09)*	0.00 (0.00–0.20)**
Blends^a^	37.00 (31.50–76.3)	120.00 (12.57–120.00)	0.24 (0.22–0.45)**	0.17 (0.10–0.25)	27.72 (19.44–56.40)	0.05 (0.03–0.09)	0.07 (0.05–0.14)	0.00 (0.00–0.26)*

*Note*: ‘Other’ categorised plant‐based beverages were not included in analysis due to inadequate nutrient composition information available for listed characterising ingredients.

^a^ Plant‐based milks comprised of two or more plant material.

** Indicates statistically significant (*p* < 0.01) difference when compared to cow's milk using a Kruskal–Wallis pairwise multiple comparisons test with Bonferroni adjustment.

* Indicates statistically significant (*p* < 0.05) difference when compared to cow's milk using a Kruskal–Wallis pairwise multiple comparisons test with Bonferroni adjustment.

No statistically significant differences were found between the micronutrient content of fortified plant‐based milks and cow's milk, although the median iodine concentration of fortified plant‐based milks was still less than 50% of that found in cow's milk (Table S1). However, unfortified plant‐based milks contained significantly less of all the respective nutrients when compared to cow's milk, except for vitamin A. Unfortified plant‐based milks also contained significantly less of each of the nutrients compared to fortified plant‐based milks.

## 4 DISCUSSION

This audit of plant‐based milks undertaken in a regional area of NSW contributes to a greater understanding of the rapidly changing Australian plant‐based milk market. The audit identified 129 plant‐based milk products and collected detailed information from each. It was revealed that fortification rates were low for nutrients other than calcium, and when compared to cow's milk, many of the plant‐based milks had significant differences in nutrient content.

The current audit identified 11% more plant‐based milk products than an Australian audit in 2020, and 39% more than an audit in 2019, demonstrating consistent growth in the market.^8^, ^28^ The three most common plant‐based milk types were overwhelmingly almond, oat and soy, making up 31%, 30% and 21% of products, respectively. Notably, Zhang et al. (2020) found only 19 grain‐based products (oat‐based plant‐based milks, and rice and quinoa beverages), compared to 39 oat beverages identified by the current audit, representing a more than two‐fold increase.^8^ These trends align with Australian market information from ‘IBISWorld’ (2023) where soy and almond milk were recognised as contributing the most in revenue to the sector, with oat milk rapidly increasing in popularity amongst consumers.^4^

The current study provides updated information on the fortification status of plant‐based milks for sale in Australia providing a comprehensive assessment quantifying the complete profile of micronutrient fortifiers in plant‐based milks. We identified that plant‐based milks are regularly fortified with calcium but not consistently with any other micronutrients. Our data confirms that there has been a consistent increase in the proportion of plant‐based milks sold in Australia that are fortified with calcium, from 42.6% in 2020 (Zhang et al.) to 79% in 2021.^13^ An opposite trend was found for fortification of plant‐based milks with vitamins D and B12. In 2020, Craig et al. identified that 21% and 35% of Australian plant‐based milks were fortified with vitamin D and vitamin B12, respectively, compared to our figures of 18% and 27%.^13^ This may be explained by differences in sampling methods, whereby Craig et al. (2021) included only plant‐based milk products sold in one major supermarket chain. Alternatively, the data may represent a shift in the plant‐based milk product landscape within recent years, with newer products being less likely to be fortified with these vitamins.

Given the significant contribution that cow's milk makes to the nutritional composition of Australian diets, it is essential to understand whether there are nutritional differences between cow's milk and plant‐based milks. The current investigation found that plant‐based milks, including both fortified and unfortified beverages, were significantly lower compared to cow's milk for protein, saturated fat, sugars, iodine, zinc, phosphorus and vitamins B12 and B2, depending on plant‐based milk type. Lower energy, protein and micronutrient content in plant‐based milk has been previously identified globally and within Australia.^1^, ^7^, ^8^, ^9^, ^10^, ^11^, ^12^, ^13^, ^14^ This has led to recommendations against plant‐based milk as an alternative for cow's milk or infant formula in Australian infant feeding guidelines.^29^, ^30^ Our audit confirmed that plant‐based milks were nutritionally dissimilar to cow's milk, supporting the notion that making a direct substitution may place vulnerable individuals, such as young children, at increased risk of nutritional inadequacy.

The current study identified many micronutrients that are significantly lower in various plant‐based milk types when compared to cow's milk, and our findings mostly align with those of Zhang et al. (2020) whose audit followed similar methodology.^8^ A point of difference was for retinol equivalents, whereby Zhang et al. (2020) reported a significantly lower concentration for all plant‐based milk categories compared to cow's milk, other than legume‐based products. Despite all plant‐based milk categories having lower median vitamin A concentrations, the current study identified a statistically significant different concentration for only oat‐based beverages. Furthermore, in the present study vitamin B12 was found to be significantly lower in all plant‐based milk categories compared to cow's milk, whereas Zhang et al. (2020) did not find a significant difference for mixed source beverages. This may be due to the addition of new ‘blended’ plant‐based milk products in recent years being less likely to be vitamin B12 fortified. To the author's knowledge, no other previous Australian research has comprehensively compared the nutritional composition of plant‐based milk to cow's milk.

All plant‐based milk categories had significantly lower concentrations of vitamin B12 when compared to cow's milk, with only 27.1% of products vitamin B12 fortified. This is concerning as those following vegetarian style eating patterns, particularly those that exclude dairy products, have significantly lower intakes of vitamin B12 and are more likely to be vitamin B12 deficient when compared to individuals that follow omnivorous eating patterns.^31^, ^32^ Importantly, previous research has revealed that vegan and vegetarians frequently supplement vitamin B12 at a rate of about 60 to 70%.^33^, ^34^ Although vegetarians and vegans participate in high rates of supplementation, reduced levels of vitamin B12 in plant‐based milk products may be contributing to inadequate vitamin B12 intakes in this group.

All plant‐based milk types except coconut, oat and rice had significantly lower concentrations of iodine compared to cow's milk, and only 3% of products identified in the audit were fortified with iodine. Adequate iodine nutrition is essential during pregnancy for foetal and maternal production of thyroid hormones required for optimal foetal neurodevelopment.^35^ Australian women of childbearing age are at increased risk of iodine deficiency despite mandatory iodine fortification of bread.^36^, ^37^, ^38^, ^39^, ^40^ The National Health and Medical Research Council guidelines recommend that pregnant and breastfeeding women, and women planning pregnancy, take a daily iodine supplement of 150 μg/day to help them meet the 220 μg/day RDI.^41^ However, this practice has not been universally adopted and pregnant women continue to have poor knowledge about iodine nutrition.^42^ Advocating for education and increased iodine fortification of plant‐based milk products will assist in supporting adequate iodine intake in this vulnerable population.

Australians have high rates of vitamin D deficiency with 36% of adults vitamin D deficient in winter.^43^ The present study identified low vitamin D fortification rates in Australian plant‐based milk products. However, due to limitations in the AUSNUT 2011–2013 database, vitamin D content of all plant‐based milk products identified in the audit could not be obtained. Additionally, no previous research has estimated vitamin D content of Australian plant‐based milks. Research globally has identified that plant‐based milks are significantly lower in vitamin D compared to cow's milk,^1^, ^9^, ^11^, ^14^ and given the prevalence of deficiency in Australia, it is vital that this be further investigated as well as fortification encouraged.

The current research identified that, in general, Australian plant‐based milks are not nutritionally equal to cow's milk. However, there was no statistically significant difference in micronutrient content in fortified plant‐based milks. Therefore, it is important that consumers be educated on nutrient differences and explore fortified plant‐based milk options, or alternative dietary sources. As the current audit identified no significant difference in cost of fortified beverages, recommending these products is reasonable. Furthermore, as fortification rates with nutrients other than calcium were low, it is recommended that plant‐based milks are fortified with more nutrients that have been identified as significantly lower in plant‐based milk compared to cow's milk. In particular, vitamin B12, iodine and vitamin D as these are at risk nutrients for some Australians.

This study provides an in‐depth analysis of the current extent of fortification and the nutritional content of plant‐based milks in the rapidly evolving Australian plant‐based milk market. Although care was taken to audit both major and minor food retailers across differing socioeconomic areas, supermarket purchases account for only 42% of plant‐based milk sales in Australia.^4^ Plant‐based milks distributed directly using a business‐to‐business supply chain model (e.g., cafés, restaurants or large‐scale food service operations such as schools and hospitals) were not included in the sampling frame. Additionally, this study relied on information provided on product packaging by manufacturers and online nutrient composition databases, rather than objective laboratory analysis of samples. This relies on the provision of an accurate ingredients list and nutritional content information provided by manufacturers. Furthermore, this study considered nutrition content and not bioavailability which may significantly impact interpretation of findings and recommendations for fortification. It is vital that bioavailability of existing nutrients and nutrient fortifiers be examined in the context of plant‐based milk to ensure effectiveness. Finally, products were only audited in food retailers geographically located in the Illawarra region of NSW and may therefore not reflect the product market of other parts of Australia.

This cross‐sectional audit of Illawarra food retailers provides updated information on plant‐based milks available for purchase. In general, and particularly due to low fortification rates for nutrients other than calcium, it was found that protein, sugar, saturated fat, iodine, phosphorus, zinc and vitamins A, B2 and B12 were significantly lower in various plant‐based milks than cow's milk. However, soy milk had comparable protein levels and there was no significant difference found between fortified beverages and cow's milk for the respective nutrient fortifier. The nutritional composition of plant‐based milks varies greatly between products and the compositions identified in the current audit indicate that Australian plant‐based milks are, in most instances, nutritionally dissimilar to cow's milk. Thus, it is important that vulnerable groups, including young children, vegans and women of childbearing age, are educated on nutritional differences, and to support this, fortification of plant‐based milk with nutrients beyond calcium is recommended.

## AUTHOR CONTRIBUTIONS

All authors contributed to the conception of the work and writing and review of the manuscript. IH undertook data collection and analysis. All authors acknowledge that the authors are in agreement with the manuscript and declare that the content has not been published elsewhere.

## CONFLICT OF INTEREST STATEMENT

The authors declare no conflicts of interest.

## Supporting information


**Table S1:** Comparison of micronutrient content of unfortified plant‐based milks, fortified plant‐based milks and cow's milks.

## Data Availability

The data that support the findings of this study are available from the corresponding author upon reasonable request.

## APPENDIX 1

### 1.1

Australian Bureau of Statistics Statistical Area Level 2 regions included in the audit:

- Albion Park‐Macquarie Pass
- Albion Park Rail
- Balgownie‐Fairy Meadow
- Berkeley‐Lake Heights‐Cringila
- Corrimal‐Tarrawanna‐Bellambi
- Dapto‐Avondale
- Figtree‐Keiraville
- Helensburgh
- Horsley‐Kembla Grange
- Kiama
- Kiama Downs‐Minnamurra
- Port Kembla‐Warrawong
- Shellharbour‐Flinders
- Shellharbour‐Oak Flats
- Thirroul‐Austinmer‐Coalcliff
- Unanderra‐Mount Kembla
- Warilla
- Windang‐Primbee
- Wollongong East
- Wollongong West
- Woonona‐Bulli‐Russel Vale

## APPENDIX 2

### 2.1

AUSNUT 2011–2013 food nutrient database food item codes utilised to represent cow's milk.^17^

- 09A10185 Milk, cow, fluid, regular fat (~3.5%)
- 09A10199 Milk, cow, fluid, skim, (~0.15% fat) added milk solids
- 09A10188 Milk, cow, fluid, skim (~0.15% fat)
- 09A10217 Milk, cow, fluid, lactose free, regular fat (~3.5%)
- 09A10218 Milk, cow, fluid, lactose free, reduced fat (~1%)
- 09A10200 Milk, cow, fluid, reduced fat (1%)
- 09A10198 Milk, cow, fluid, reduced fat (1.5%), added omega 3 polyunsaturates
