# Supplementary material for: How do plant‐based milks compare to cow's milk nutritionally? An audit of the plant‐based milk products available in Australia
Source: Nutr Diet. 2024 Sep 29;82(1):76–85. doi: 10.1111/1747-0080.12906 (PMC11795225; doi:10.1111/1747-0080.12906)
Supplement: Supplementary file 1 — Table S1: Comparison of micronutrient content of unfortified plant‐based milks, fortified plant‐based milks and cow's milks. [file NDI-82-76-s001.docx]

Supplementary Table 1: Comparison of micronutrient content of unfortified plant-based milks, fortified plant-based milks and cow’s milks.

|  | **Cow’s Milk** | **Unfortified plant-based milk^a^** | **Fortified plant-based milk^a^** |
| --- | --- | --- | --- |
| **Nutrient** | Median (Q1-Q3) | Median (Q1-Q3) | Median (Q1-Q3) |
| **Iodine (ug/100mL)** *,** | 22.30  (14.40-25.70) | 0.24  (0.21-0.55) | 7.50  (7.50-7.50) |
| **Calcium (mg/100mL)** *,** | 118.00  (110.00-122.00) | 6.55  (4.33-11.26) | 120 .00  (104.00-120.00) |
| **Vitamin A (ug/100mL)** ** | 12.50  (0.01-41.75) | 0.05  (0.00-0.08) | 43.00  (40.00-50.00) |
| **Vitamin B2 (mg/100mL)** *,** | 0.20  (0.18-0.20) | 0.02  (0.00-0.04) | 0.17  (0.17-0.20) |
| **Vitamin B12 (ug/100mL)** *,** | 0.60  (0.60-0.60) | 0.00  (0.00-0.00) | 0.40  (0.30-0.40) |
| **Phosphorus (mg/100mL)** *,** | 96.00  (88.00-98.00) | 33.60  (15.84-51.00) | 100.00  (100.00-102.50) |
| **Vitamin D (mg/100mL)** | n/a^b^ | n/a^b^ | 0.50  (0.50-0.50)^c^ |

*Indicates significant (p<0.01) difference between unfortified plant-based milk and cow’s milk using a Kruskal-Wallis pairwise multiple comparisons test with Bonferroni adjustment.

**Indicates a significant (p<0.01) difference between fortified and unfortified plant-based milk using a Kruskal-Wallis pairwise multiple comparisons test with Bonferroni adjustment.

^a^Includes all types of plant-based milk.

^b^Information unavailable in AUSNUT 2011-13 food composition database.

^c^Value extracted from product nutrition information panels.
